# Human Single-chain Variable Fragments Neutralize *Pseudomonas aeruginosa* Quorum Sensing Molecule, 3O-C12-HSL, and Prevent Cells From the HSL-mediated Apoptosis

**DOI:** 10.3389/fmicb.2020.01172

**Published:** 2020-06-24

**Authors:** Sirijan Santajit, Watee Seesuay, Kodchakorn Mahasongkram, Nitat Sookrung, Pornpan Pumirat, Sumate Ampawong, Onrapak Reamtong, Manas Chongsa-Nguan, Wanpen Chaicumpa, Nitaya Indrawattana

**Affiliations:** ^1^Department of Microbiology and Immunology, Faculty of Tropical Medicine, Mahidol University, Bangkok, Thailand; ^2^Center of Research Excellence on Therapeutic Proteins and Antibody Engineering, Department of Parasitology, Faculty of Medicine Siriraj Hospital, Mahidol University, Bangkok, Thailand; ^3^Biomedical Research Unit, Department of Research, Faculty of Medicine Siriraj Hospital, Mahidol University, Bangkok, Thailand; ^4^Department of Tropical Pathology, Faculty of Tropical Medicine, Mahidol University, Bangkok, Thailand; ^5^Department of Tropical Molecular Biology and Genetics, Faculty of Tropical Medicine, Mahidol University, Bangkok, Thailand; ^6^Faculty of Public Health and Environment, Pathumthani University, Pathum Thani, Thailand

**Keywords:** *Pseudomonas aeruginosa*, quorum sensing, *N*-3-oxo-dodecanoyl-*L*-homoserine lactone (3O-C12-HSL), apoptosis, human scFv

## Abstract

The quorum sensing (QS) signaling molecule, *N*-(3-oxododecanoyl)-*L*-homoserine lactone (3O-C12-HSL), contributes to the pathogenesis of *Pseudomonas aeruginosa* by regulating expression of the bacterial virulence factors that cause intense inflammation and toxicity in the infected host. As such, the QS molecule is an attractive therapeutic target for direct-acting inhibitors. Several substances, both synthetic and naturally derived products, have shown effectiveness against detrimental 3O-C12-HSL activity. Unfortunately, these compounds are relatively toxic to mammalian cells, which limits their clinical application. In this study, fully human single-chain variable fragments (HuscFvs) that bind to *P. aeruginosa* haptenic 3O-C12-HSL were generated based on the principle of antibody polyspecificity and molecular mimicry of antigenic molecules. The HuscFvs neutralized 3O-C12-HSL activity and prevented mammalian cells from the HSL-mediated apoptosis, as observed by Annexin V/PI staining assay, sub-G1 arrest population investigation, transmission electron microscopy for ultrastructural morphology of mitochondria, and confocal microscopy for nuclear condensation and DNA fragmentation. Computerized homology modeling and intermolecular docking predicted that the effective HuscFvs interacted with several regions of the bacterially derived ligand that possibly conferred neutralizing activity. The effective HuscFvs should be tested further *in vitro* on *P. aeruginosa* phenotypes as well as *in vivo* as a sole or adjunctive therapeutic agent against *P. aeruginosa* infections, especially in antibiotic-resistant cases.

## Introduction

*Pseudomonas aeruginosa*, a versatile and ubiquitous Gram-negative bacterium, is an opportunistic microorganism that frequently causes severe nosocomial infections, particularly among immunocompromised patients and those suffering from cystic fibrosis, burns, HIV infection, and cancer (Tang et al., 1996; Sadikot et al., 2005; Silva Filho et al., 2013; Malhotra et al., 2019; Waters and Goldberg, 2019). The pathogenicity of *P. aeruginosa* is attributable mainly, if not solely, to the regulons of two complete *N*-acyl homoserine lactone (AHL)-dependent quorum sensing (QS) systems, called LasI/R and RhlI/R (Preston et al., 1997; Venturi, 2006). The two QS systems act in a hierarchical manner, i.e., the lasI/R system controls the activity of the rhlI/R circuit (Pearson et al., 1994, 1995). During bacterial infection, the LasI and RhlI synthases produce *N*-(3-oxododecanoyl)-*L*-homoserine lactone (3O-C12-HSL) and *N*-butanoyl-*L*-homoserine lactone (C4-HSL), respectively. The QS molecules then interact with their cognate LasR and RhlR, causing transcription of hundreds of target genes, including those coding for virulence factors such as lectins, elastases, proteases, exotoxin A, pyocyanin, and surface-active rhamnolipids important in the late stages of biofilm development, as well as genes involved in antibiotic resistance (Wagner et al., 2003; Venturi, 2006; Rutherford and Bassler, 2012; Moradali et al., 2017).

*N*-(3-Oxododecanoyl)-*L*-homoserine lactone (3O-C12-HSL) is the prominent autoinducer of the *P. aeruginosa* QS system (Duan and Surette, 2007; Rasamiravaka and El Jaziri, 2016). 3O-C12-HSL is a small, fatty acid-like, membrane-permeant signaling molecule that comprises a hydrophilic homoserine lactone ring linked to the hydrophobic 12-carbon-atom-long acyl side chain via an amide bond (Eberhard et al., 1981; Pearson et al., 1995; Ritchie et al., 2007; O’Connor et al., 2015). The roles of 3O-C12-HSL in pathogenesis and modulation of the host immune responses have been reviewed (Liu et al., 2015). Owing to its lipophilicity, the 3O-C12-HSL can traverse the mammalian cell membrane (Ritchie et al., 2007), causing mitochondrial damage and dysfunction, which subsequently activates the caspase pathway leading to apoptosis of several cell types, including macrophages, neutrophils, T lymphocytes, human vascular endothelial cells, murine fibroblasts, airway epithelial cells, goblet cells, and breast carcinoma cells (Tateda et al., 2003; Li et al., 2004; Shiner et al., 2006; Jacobi et al., 2009; Schwarzer et al., 2012; Tao et al., 2016, 2018). *P. aeruginosa* QS signaling molecules also modulate host immune responses by down-regulating the expression of co-stimulatory molecules on dendritic cells (DCs), leading to inhibition of DC maturation and their ability to activate effector T-cell responses (Boontham et al., 2008). Because the 3O-C12-HSL plays an important role in the virulence and pathogenesis of *P. aeruginosa* and host immunity suppression, it is an attractive target for novel therapeutics for *P. aeruginosa* infection. Substances that interfere with *P. aeruginosa* 3O-C12-HSL activity should mitigate bacterial-associated disease severity, although blocking the QS system alone does not necessarily abrogate all *P. aeruginosa* virulence factors, such as T3SS (Bleves et al., 2005; López-Jácome et al., 2019; Soto-Aceves et al., 2019). A therapeutic approach based on QS interference and/or attenuation of QS signals should result in greater sensitivity of the *P. aeruginosa* to stresses, such as antimicrobial drugs (Rasmussen and Givskov, 2006; Defoirdt et al., 2010; Maeda et al., 2012; Kalia et al., 2014; Krzyżek, 2019).

Recently, a murine monoclonal antibody (mAb), RS2-1G9, against a lactam mimetic of 3O-C12-HSL has been shown to prevent apoptosis through p38 mitogen-activated protein kinase activation and protected murine bone marrow-derived macrophages from the cytotoxic effects of the QS molecule (Kaufmann et al., 2006, 2008). The RS2-1G9 paratope was shown to enclose the polar lactam moiety of the 3O-C12-HSL molecule in the co-crystal structure of the Fab fragment of the RS2-1G9 mAb and the target 3O-C12-HSL completely (Debler et al., 2007). Active immunization of mice with 3O-C12-HSL-protein conjugate protected immunized mice from lethal *P. aeruginosa* infection (Miyairi et al., 2006). Antibody-based therapy directed to the QS molecule should not only block bacterial virulence, but also rescue the host immunity that had been modulated/suppressed by the QS system (Kaufmann et al., 2008; Palliyil and Broadbent, 2009). The present study generated engineered, fully human, single-chain antibody variable fragments (HuscFvs) that neutralize 3O-C12-HSL bioactivity. The HuscFvs should be tested, step-by-step, toward clinical application as a sole or adjunct therapy for the currently failing antibiotic treatment of patients with *P. aeruginosa* infection.

## Materials and Methods

### *P. aeruginosa N*-(3-Oxododecanoyl)-*L*-Homoserine Lactone (3O-C12-HSL)

The QS molecule was synthesized commercially (Cayman Chemical, Ann Arbor, MI, United States) under the IUPAC name: 3-oxo-*N*-[(3*S*)-2-oxooxolan-3-yl]-dodecanamide. 3O-C12-HSL was stored in 100% dimethyl-sulfoxide (DMSO) and diluted with phosphate-buffered saline, pH 7.4 (PBS), to the desired concentration for use.

### Preparation of HuscFv to *P. aeruginosa* 3O-C12-HSL

The human single-chain variable fragments (HuscFvs) to the 3O-C12-HSL were generated based on the principles of the polyspecific property of an antibody, i.e., one antibody can bind different antigens by paratope adaptation to accommodate distinct antigens, such as through differential engagements of the complementarity determining regions (CDRs), and the molecular mimicry of the antigens (different antigens can share surface topologies in terms of shape or chemical nature) (Tapryal et al., 2013). In this study, HB2151 *Escherichia coli* clones carrying phagemids with inserted HuscFv genes (*huscfvs*) were previously selected from a HuscFv phage display library (Kulkeaw et al., 2009) using *Pseudomonas* exotoxin A (ETA) as antigen in the phage-biopanning process (Santajit et al., 2019). Genes coding for HuscFvs of individual *E. coli* clones were sequenced and deduced, and the canonical CDRs and framework regions (FRs) of both VH and VL domains were determined based on the numbering scheme of Chotia and Kobat (Abhinandan and Martin, 2008).

Three dimensional (3D) models of the selected HuscFvs were generated by subjecting their deduced amino acid sequences to the I-TASSER online server (Yang et al., 2015). The HuscFvs-3D models from the I-TASSER were further refined to improve local geometric and physical quality using ModRefiner (Xu and Zhang, 2011). The quality of the generated homology models of HuscFvs was then evaluated using the PROCHECK server to provide Ramachandran plots (Laskowski et al., 1993). Thereafter, the 3D structures of the individual HuscFvs were superimposed with the 3D structure of the mAb RS2-1G9 F(ab′)_2_ (PDB ID: 2NTF) (previously shown to neutralize 3O-C12-HSL pathogenic activity; hence, the mAb has been designated as a “quorum quenching antibody”) (Kaufmann et al., 2006, 2008) using the CLICK server, i.e., the topology-independent tool comparing 3D structures without a scoring function measuring structural similarity (Nguyen et al., 2011). The HuscFvs showing top-scored topological similarity with the RS2-1G9 antigen-binding site were selected. The 3D structure of 3O-C12-HSL was retrieved from the PubChem database, a resource of chemical molecules and their bioactivities (PubChem CID: 3246941) (Kim et al., 2015). The modeled-3O-C12-HSL F(ab′)_2_ was docked with the 3D model of each HuscFv receptor binding pocket using Autodock Vina software (Trott and Olson, 2010; Forli et al., 2016). The conformation of each HuscFv-ligand complex with the lowest binding free energy (ΔG) at the best docking position was selected for interaction analysis and visualization through the Discovery studio visualizer 3.5 program.

### Large-Scale Production of HuscFvs

The *E. coli* clones carrying phagemids containing the DNA coding for the selected HuscFvs were subjected to sub-cloning for large scale HuscFv production. The *huscfvs* were PCR-amplified from the *huscfv*-pCANTAB5E phagemids of HB2151 *E. coli* clones using a Phusion High-Fidelity DNA polymerase (Thermo Fisher Scientific, Carlsbad, CA, United States). The PCR specific primers were forward-*huscfv*-LIC: 5′-GGTTGGGAATTGCAAGCGGC CCAGCCGGCC-3′ and reverse-*E-tag*-LIC: 5′-GGAGATGGGA AGTCATTAACGCGGTTCCAGCGGATCC-3′. The *huscfv* inserts were designed to consist of a HuscFv-coding sequence linked to specific sequences for ligation independent cloning (LIC) protocol (Thermo Fisher Scientific). The amplified *huscfv-E-tag* DNAs were cloned separately into the pLATE52 vector (Thermo Fisher Scientific). Recombinant pLATE52-*huscfv* plasmids were transformed into JM109 *E. coli* by the heat-shock method. After PCR screening and DNA sequencing, the recombinant plasmids were introduced into an expression host, NiCo21(DE3) *E. coli* (New England Biolabs, St. Albans, Herts, United Kingdom), and the transformed bacteria were grown at 37°C for 16 h on LB agar containing 100 μg/ml of ampicillin. A single colony of each transformed clone was cultured in LB broth containing 100 μg/ml ampicillin with shaking (250 rpm) at 37°C for 16 h. The overnight cultures (12.5 ml) were separately inoculated into the fresh LB medium (250 ml) containing ampicillin and grown at 37°C until an OD_600 nm_ reached ∼0.6–0.8. Recombinant HuscFv expression was induced by adding isopropyl-β*-D*-1-thiogalactopyranoside (IPTG) to a final concentration of 1 mM and incubated at 30°C for 6 h. The bacterial pellets were collected by centrifugation at 5,000 × *g* at 4°C for 20 min.

The recombinant HuscFvs were purified from the bacterial inclusion bodies (IBs) as described previously (Jittavisutthikul et al., 2016). Two grams of *E. coli* wet cell pellets were resuspended in 10 ml of BugBuster^TM^ protein extraction reagent (Novagen, Schwalbach, Germany) and 20 μl of Lysonase^TM^ bioprocessing reagent (Novagen) were added to each preparation. The preparations were kept at 25°C on a rotator for 20 min and cell pellets were collected after centrifugation at 8,000 × *g* at 4°C for 30 min. The IBs were washed with Wash-100 reagent [50 mM sodium phosphate buffer, pH 8.0; 500 mM NaCl; 5 mM EDTA; 8% (w/v) glycerol; and 1% (v/v) Triton X-100] twice and once with Wash-114 buffer [50 mM Tris–HCl, pH 8.0; 300 mM NaCl; and 1% (v/v) Triton X-114] with shaking at high speed for 40 min, and the IB pellets were then collected. The IBs were then washed with Wash-Solvent solution [50 mM Tris–HCl, pH 8.0; and 60% (v/v) isopropanol] and sterile ultrapure distilled water on ice, also with vigorous shaking, and centrifuged. Thereafter, 2.5 mg of purified IB pellets were solubilized in 5 ml of solubilizing buffer [50 mM CAPS, pH 11.0; 0.3% (w/v) *N*-lauryl sarcosine; and 1 mM dithiothreitol (DTT)] and kept at 4°C for 16 h. After dissolving completely, the protein was loaded into Snakeskin dialysis tubing with a molecular weight cut-off of 10 kDa (Thermo Fisher Scientific), and dialyzed against 750 ml of refolding buffer (20 mM imidazole, pH 8.5, supplemented with 0.1 mM DTT) at 4°C with slow stirring. After 3 h, the buffer was changed to a fresh refolding buffer, and dialysis was continued for 16 h. The refolded protein was subsequently dialyzed against a dialysis buffer without DTT with slow stirring at 4°C for 16 h. Each preparation was filtered through a 0.2-μm low protein binding Acrodisc^®^ Syringe Filter (Pall, Port Washington, NY, United States) and kept at 30°C in a water bath for 3 h before adding 60 mM trehalose. The protein concentration of the refolded HuscFvs was determined using Pierce^®^ BCA Protein Assay, while the quality and purity of the recombinant proteins were analyzed by SDS-PAGE and stained with Coomassie Brilliant Blue G-250 (Bio-rad, Hercules, CA, United States). Refolded HuscFv preparations were concentrated using Amicon^®^ Ultra 4 ml 3K centrifugal filter devices (Merck Millipore, Darmstadt, Germany) and stored at −20°C until use.

### Circular Dichroism

The buffer of the HuscFv preparations was changed to 20 mM sodium phosphate buffer, pH 8.5, at a protein concentration of 0.1 mg/ml, and the antibodies were subjected to CD measurement. The data were recorded using a JASCO spectrometer (model J-815) equipped with a Peltier temperature controller system (Jasco, Tokyo, Japan) in a 1 mm path-length quartz cuvette. The proteins were scanned at 50 nm/min at 25°C. The CD spectra were collected over a wavelength range of 190–260 nm.

### Cell Line

Human cervical carcinoma, HeLa, cells were cultured in Dulbecco’s Modified Eagle’s Medium (DMEM; Gibco, Carlsbad, CA, United States) supplemented with 10% (v/v) fetal bovine serum (Hyclone, Novato, CA, United States) and 1% (v/v) penicillin-streptomycin (complete DMEM) at 37°C in a 5% CO_2_ atmosphere.

### Determination of the Biocompatibility of the HuscFvs to Mammalian Cells

The monolayer of HeLa cells established in individual wells of a 24-well tissue culture plate (∼2 × 10^5^ cells/well) were washed with sterile PBS, added with 2 μM of individual HuscFv preparations in complete DMEM, and incubated at 37°C in 5% CO_2_ atmosphere for 24 h. Cells in the medium alone served as a background control. After 24 h, the percent viability of cells of each treatment was analyzed using an FITC-Annexin V Apoptosis Detection Kit (BD Biosciences, San Jose, CA, United States) according to the manufacturer’s protocols. The cells were washed with Dulbecco’s phosphate-buffered saline (DPBS) and resuspended in binding buffer. Five microliters of Annexin V-FITC conjugate and 5 μl of propidium iodide (PI) were added. After 15-min incubation at room temperature (25°C) in darkness, apoptotic cells were enumerated by flow cytometric analysis (BD LSRFortessa^TM^, San Jose, CA, United States) using BD FACSDiva^TM^ software (BD Biosciences). At least 20,000 events of single cells per sample were collected.

### Cellular Apoptosis Mediated by 3O-C12-HSL

HeLa cells (∼2 × 10^5^ cells/well) were treated with various concentrations of 3O-C12-HSL, i.e., 10, 25, 50, 75, and 100 μM. The background control comprised of cells incubated with medium alone. After incubation at 37°C in a CO_2_ incubator for 18 h, the cells were harvested and subjected to Annexin V/PI binding assay, as described above.

### Neutralization of 3O-C12-HSL-mediated-Cytotoxicity by HuscFvs

Fifty micromolars of 3O-C12-HSL in 0.25% DMSO were mixed with various concentrations of individual HuscFv preparations (0.25, 0.5, 1.0, and 1.2 μM) for 1 h before adding to HeLa cells (∼2 × 10^5^ cells/well) and incubated at 37°C in a CO_2_ incubator for 18 h. After incubation, the cells were collected, washed, double-stained with Annexin V-FITC and PI, and analyzed by flow cytometry, as described above. Three independent experiments were performed.

### Neutralization of 3O-C12-HSL-mediated Cell Cycle Arrest by HuscFvs

HeLa cells (∼2 × 10^5^ cells/well) were treated with a mixture of 50 μM 3O-C12-HSL and 1 μM of individual HuscFvs for 18 h. HeLa cells exposed to medium alone served as a control. After incubation, cells were washed with ice-cold PBS, fixed in 70% ethanol, and kept at −20°C overnight. Cells were then washed 3 times with ice-cold PBS and incubated in 500 μl of stain solution [10 μg/ml PI, 100 μg/ml RNase, and 0.1% (v/v) Triton X-100 in DPBS, pH 7.4] at room temperature in darkness for 30 min. The DNA contents of the cells were measured, and cell cycle histograms/distributions were generated. Then, the percentage of cells in the sub-G1 phase was determined by flow cytometry (BD LSRFortessa^TM^) using BD FACSDiva^TM^ software (BD Biosciences), with at least 10,000 recorded events per sample.

### Analysis of Nuclear Damage by Fluorescence Staining

Nuclear damage was studied using 4’,6-diamidino-2-phenylindole (DAPI) staining. Briefly, HeLa cells (1 × 10^6^ cells) were seeded on a 22 × 22 mm square coverslip (Menzel-Glaser, Braunschweig, Germany) in a 6-well plate (Costar, New York, NY, United States) and kept at 37°C in a 5% CO_2_ incubator for 24 h. The culture medium was removed and the cells were replenished with complete DMEM containing a mixture of 3O-C12-HSL (50 μM) and HuscFvs (2 μM). After 18 h, the cells were washed and fixed with 4% (v/v) paraformaldehyde in PBS, permeabilized with 1% (v/v) Triton X-100 in PBS, blocked with 3% (w/v) bovine serum albumin (BSA) in PBS at room temperature for 30 min, then washed. The permeabilized cells were stained and mounted with DAPI (1:5,000) (Molecular Probes, Carlsbad, CA, United States) in the anti-fade mounting medium. DNA fragmentation and chromatin condensation were observed under a confocal microscope (Carl Zeiss Laser Scanning System LSM 700, Jena, Germany). Images were processed using the Zeiss LSM Image Browser (version 6.0.0.309).

### Transmission Electron Microscopy

Transmission electron microscopy (TEM) was used to examine the ultrastructural changes of the HeLa cell mitochondria after various treatments. The cells from each treatment group were fixed with 2.5% (v/v) glutaraldehyde in 0.1 M sucrose phosphate buffer (SPB) at room temperature for 1 h, washed three times with 0.1 M SPB, post-fixed with 1.0% (w/v) osmium tetroxide in the same buffer for 1 h, and dehydrated with a graded series of ethanol. The dehydrated cells were infiltrated with pure LR white embedded medium (EMS^®^, Hatfield, PA, United States) in 70% (v/v) ethanol, embedded in a capsule beam, and incubated at 65°C for 48 h. The ultrathin (100 nm) sections of the cells were prepared; the sections were positioned on a 200 square-mesh copper grid and stained with ethanolic uranyl acetate and lead citrate. The morphological and structural characteristics of mitochondria were observed under a transmission electron microscope (model HT7700, Hitachi, Tokyo, Japan).

### Statistical Analysis

Statistical analyses of all experiments were performed using GraphPad Prism 5 software (La Jolla, CA, United States). One-way ANOVA followed by Tukey’s *post hoc* multiple comparison tests were used to analyze the differences between groups. All data are shown as mean ± SD. Statistically significant difference was set at *p* < 0.05.

## Results

### HuscFvs to 3O-C12-HSL

The refined models of HuscFvs for the selected HB2151 *E. coli* clones derived from phage biopanning with *P. aeruginosa* exotoxin A revealed that HuscFvs of three *E. coli* clones, i.e., E44 (HuscFv-E44), F15 (HuscFv-F15), and F19 (HuscFv-F19), showed reliable Ramachandran plots. The percent residues in the most favored regions, the additional allowed regions, the generously allowed regions, and the disallowed regions of the Ramachandran diagrams of the HuscFv-E44, HuscFv-F15, and HuscFv-F19 were 90.1, 6.8, 1.0, and 2.1 %; 91.3, 7.7, 0.5, and 0.5%; and 88.2, 9.4, 1.0, and 1.5%, respectively (Supplementary Figure S1).

**FIGURE 1:**
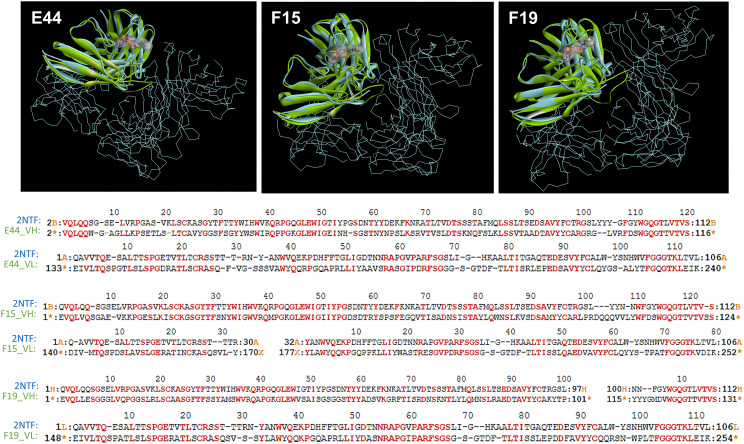
The 3D complex of RS2-1G9 F(ab′)_2_ fragment and *N*-Acyl-*L*-homoserine lactone analog (PDB:2NTF) was superimposed by the HuscFv-E44 (left), HuscFv-F15 (middle), and HuscFv-F19 (right) using CLICK: http://mspc.bii.a-star.edu.sg/click **(Upper panel)**. One antigen-binding site of the mAb RS2-1G9 F(ab′)_2_ fragment (VH and VL domains, shown in blue) was superimposed by the HuscFvs (green). The trace illustration is the remaining portion of the RS2-1G9 F(ab′)_2_. **Lower panel**, the superimposed amino acids of the RS2-1G9 antigen-binding site (2NTF) and the VH and VL of HuscFv-E44, HuscFv-F15, and HuscFv-F19, are shown in red alphabets.

From structural comparisons of individual HuscFvs with the antigen-binding site of the well-characterized quorum quenching mAb, i.e., RS2-1G9 (shown previously to bind to and neutralize the activities of *P. aeruginosa* 3O-C12-HSL), it was found that the binding pockets of the three HuscFvs were superimposed with the antigen-binding site of RS2-1G9. The coverage percentages of the overlapping structures between the modeled HuscFv-E44, HuscFv-F15, and HuscFv-F19 and the RS2-1G9 were 90.83, 89.29, and 88.58%, respectively (Figure 1 and Supplementary Table S1).

### Homology Modeling and Intermolecular Docking Between HuscFvs and 3O-C12-HSL

**FIGURE 2:**
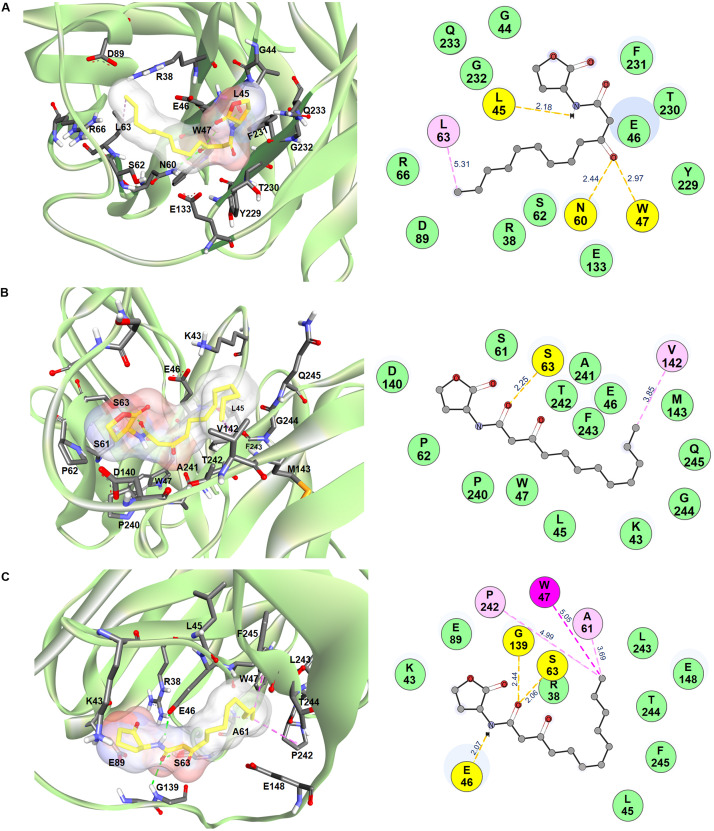
Computerized contact interfaces between 3O-C12-HSL and HuscFvs. **(A–C)** on the left side of the panels show interaction between 3D structures of 3O-C12-HSL (yellow stick) and green ribbons of **(A)** HuscFv-E44, **(B)** HuscFv-F15, **(C)** HuscFv-F19. Right side of the panels **(A–C)** show residues of the respective HuscFvs and the HuscFv-3O-C12-HSL interactive bonds (yellow, hydrogen bond; light pink, alkyl; magenta, Pi-alkyl; green, van der Waals force).

*In silico* intermolecular docking was performed to investigate the interaction of the HuscFvs with the 3O-C12-HSL. The residues of HuscFv-E44, HuscFv-F15, and HuscFv-F19 that tentatively formed interactive bonds with the haptenic 3O-C12-HSL target are shown in Figure 2 and Table 1. The Gibbs free energy (ΔG) of the representative complexes of respective HuscFvs with the ligand were −5.6, −5.8, and −5.4 kcal/mol, respectively.

**TABLE 1:** Residues of *Pseudomonas aeruginosa* 3O-C12-HSL predicted to form contact interfaces with the effective HuscFv-E44, HuscFv-F15, and HuscFv-F19.

3O-C12-HSL position	HuscFv-E44		Interactive bond
	Residue (s)	Domain	
Ketone group of HSL ring	E46	VH-FR2	Van de Waals
C3 of HSL ring	G44 G232	VH-FR2 VL-FR4	Van de Waals Van de Waals
C4 of HSL ring	G44	VH-FR2	Van de Waals
NH-group	L45 E46 F231	VH-FR2 VH-FR2 VL-FR4	Hydrogen Van de Waals Van de Waals
1-oxo-group	T230 F231	VL-CDR3 VL-FR4	Van de Waals Van de Waals
C2 of acyl chain	E46 T230	VH-FR2 VL-CDR3	Van de Waals Van de Waals
3-oxo-group	W47 N60	VH-FR2 VH-CDR2	Hydrogen Hydrogen
	E46 Y229	VH-FR2 VL-CDR3	Van de Waals Van de Waals
C5 of acyl chain	E133	VL-FR1	Van de Waals
C9 of acyl chain	S62	VH-CDR2	Van de Waals
C10, C11 of acyl chain	R38 R66	VH-FR2 VH-FR3	Van de Waals Van de Waals
C12 of acyl side chain	L63	VH-CDR2	Hydrophobic (alkyl)
C12 of acyl side chain	R38 R66 N89	VH-FR2 VH-FR3 VH-FR3	Van de Waals Van de Waals Van de Waals

**3O-C12-HSL position**	**HuscFv-F15**		**Interactive bond**
	**Residue(s)**	**Domain**	

C3 of HSL ring	P62	VH-CDR2	Van de Waals
C3, C4 of HSL ring	D140	VL-FR1	Van de Waals
1-oxo-group	W47 S61	VH-FR2 VH-CDR2	Van de Waals Van de Waals
	S63	VH-CDR2	Hydrogen
C2 of acyl chain	W47/A241	VH-FR2	Van de Waals
3-oxo-group	P240/A241/T242	VL-CDR3	Van de Waals
	W47 A241	VH-FR2 VL-CDR3	Van de Waals Van de Waals
C4 of acyl chain	W4 F243	VH-FR2 VL-FR4	Van de Waals Van de Waals
	A241/T242	VL-CDR3	Van de Waals
C5 of acyl chain	E46, W47 F243	VH-FR2 VL-FR4	Van de Waals Van de Waals
C6 of acyl chain	F243	VL-FR4	Van de Waals
C7 of acyl chain	L45, E46 F243	VH-FR2 VL-FR4	Van de Waals Van de Waals
C8 of acyl chain	K43, E46	VH-FR2	Van de Waals
C9 of acyl chain	K43	VH-FR2	Van de Waals
	F243/G244/Q245	VL-FR4	Van de Waals
C10 of acyl chain	F243/G244/Q245	VL-FR4	Van de Waals
C12 of acyl chain	V142	VL-FR1	Hydrophobic (alkyl)
	M143 T242 F243	VL-FR1 VL-CDR3 VL-FR4	Van de Waals Van de Waals Van de Waals

**3O-C12-HSL position**	**HuscFv-F19**		**Interactive bond**
	**Residue(s)**	**Domain**	

Ketone group of HSL ring	R38 E89	VH-FR2 VH-FR3	Van de Waals Van de Waals
C3, C4 of HSL ring	K43	VH-FR2	Van de Waals
Oxygen of HSL ring	E89	VH-FR3	Van de Waals
NH-group	R38, E46	VH-FR2	Hydrogen
1-oxo-group	R38	VH-FR2	Van de Waals
	S63 G139	VH-CDR2 Linker	Hydrogen Hydrogen
C2 of acyl side chain	R38	VH-FR2	Van de Waals
C7, C8 of acyl chain	L45 F245	VH-FR2 VL-FR4	Van de Waals Van de Waals
C9 of acyl side chain	T244 F245	VL-CDR3 VL-FR4	Van de Waals Van de Waals
C10 of acyl chain	E148	VL-FR1	Van de Waals
C11 of acyl chain	L243	VL-CDR3	Van de Waals
C12 of acyl chain	W47 A61 P242	VH-FR2 VH-CDR2 VL-CDR3	Hydrophobic (π-alkyl) Hydrophobic (alkyl) Hydrophobic (alkyl)

The HuscFv-E44 used residues from VH-CDR2 and VL-CDR3, as well as help from VH-FR2, VH-FR3, VL-FR1, and VL-FR4 to form contact interfaces with the functional groups of 3O-C12-HSL. The interactions were three hydrogen bonds between L45 of VH-FR2 with the NH group of the coordinated 3O-C12-HSL (2.18 Å) and N60 of VH-CDR2, and W47 of VH-FR2 with the 3O-C12-HSL carbonyl oxygen of 3-oxo-group of the acyl chain (2.97 and 2.44 Å, respectively). There was one hydrophobic interaction (alkyl) formed between L63 of VH-CDR2 and C12 of the acyl chain of AHL. HuscFv-E44 also used many residues in different domains to form contact interfaces via van der Waals forces with the 3O-C12-HSL, including S62 of VH-CDR2; Y229 and T230 of VL-CDR3; R38, G44, and E46 of VH-FR2; R66 and D89 of VH-FR3, E133 of VL-FR1; and F231, G232, and Q233 of VL-FR4 (Table 1).

HuscFv-F15 formed a hydrogen bond (2.25 Å) with the carbonyl oxygen of the 1-oxo-group of the fatty acid-like ligand through S63 of VH-CDR2. This antibody also used V142 of VL-FR1 to form hydrophobic contact (alkyl) with the C12 of the hapten acyl chain. Several other positions of the 3O-C12-HSL molecule have interacted via van der Waals forces with several residues of the HuscFv-F15 including S61 and P62 of VH-CDR2; P240, A241, and T242 of VL-CDR3; K43, L45, E46, and W47 of VH-FR2; D140 and M143 of VL-FR1; and F243, G244, and Q245 of VL-FR4.

Serine 63 of VH-CDR2 and G139 of the HuscFv-F19 linker formed contact with the carbonyl oxygen of the 1-oxo-group of the 3O-C12-HSL via hydrogen bonds (2.06 and 2.44 Å, respectively). Hydrogen bonding also occurred between E46 of VH-FR2 and the NH-group of the HSL backbone (2.07 Å). The last carbon atom of the long acyl side chain of the 3O-C12-HSL formed π-alkyl hydrophobic interaction with W47 of VH-FR2 as well as the alkyl hydrophobic interaction with A61 of VH-CDR2 and P242 of VL-CDR3. In addition, the HuscFv-F19 formed van der Waals contacts with the 3O-C12-HSL by using L243 and T244 of VL-CDR3; R38, K43, and L45 of VH-FR2; E89 of VH-FR3; E148 of VL-FR1; and F245 of VL-FR4.

The results of the structural comparison of the HuscFvs with the quorum quenching mAb, RS2-1G9, and the intermolecular docking between the HuscFvs and the 3O-C12-HSL enticed us to test further the ability of HuscFvs to neutralize *P. aeruginosa* 3O-C12-HSL activities.

### Large-Scale Production of HuscFvs

**FIGURE 3:**
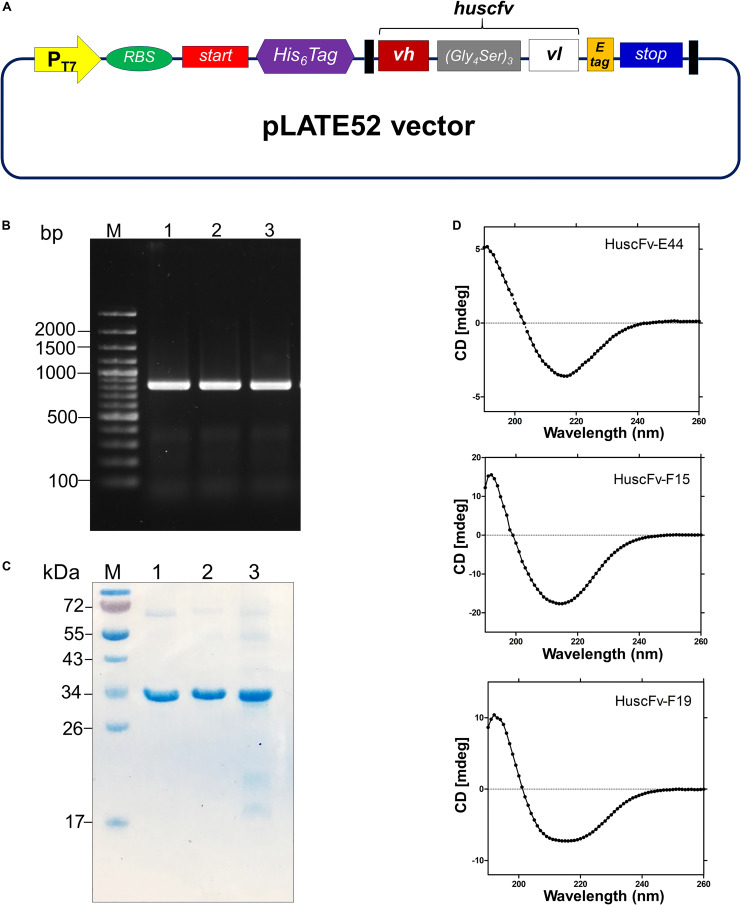
Production and characterization of HuscFvs to 3O-C12-HSL. **(A)** Schematic diagram of the inserted DNA construct in pLATE52 where the DNA sequence coding for HuscFv (*vh-linker-vl*) was flanked with DNA sequences of 6 × His at the 5′ end and E-tag at the 3′ end. **(B)** Amplicons of *huscfv*-LIC fragments (∼ 850 bp) for sub-cloning into pLATE52 vector. M, 100 bp-plus DNA ladder; 1–3, *huscfv*-LIC amplicons of three representatives transformed NiCo21(DE3) *E. coli* clones. Numbers at the left are DNA sizes in bp. **(C)** Stained SDS-PAGE-separated purified recombinant HuscFvs. M, protein standard; 1–3, purified HuscFv-E44, HuscFv-F15 and HuscFv-F19, respectively. Numbers at the left are protein masses in kDa. **(D)** CD spectra of the refolded HuscFv-E44, HuscFv-F15, and HuscFv-F19.

The *huscfv* inserts in the pCANTAB5E phagemids of the *E. coli* clones E44, F15, and F19 were sub-cloned into the pLATE52 vector. The DNA construct in the vector is shown in Figure 3A. The amplicon of DNA coding for 6 × His tagged-HuscFv formed a PCR amplicon band at ∼ 850 bp, as revealed on agarose gel (Figure 3B). The refolded and purified HuscFv-E44, HuscFv-F15, and HuscFv-F19, with molecular sizes of about 34 kDa, are shown in Figure 3C.

Secondary structures of the refolded HuscFvs were determined by far-UV CD spectroscopy. The far-UV CD spectra (190–260 nm) for all HuscFvs revealed their β-sheet structures, which shared a similar CD spectra pattern (Figure 3D). The antibody preparations did not form aggregates.

### Biocompatibility of HuscFvs to Mammalian Cells

HeLa cells exposed to 2 μM of HuscFv-E44, HuscFv-F15, and HuscFv-F19 for 24 h showed more than 90% viability, which was not different from the cells in medium alone (*p* > 0.05) (Supplementary Figure S2) indicating biocompatibility of the HuscFvs to the representative mammalian cells.

### HuscFvs-bound 3O-C12-HSL Had Impairment in Inducing Mammalian Cell Apoptosis

**FIGURE 4:**
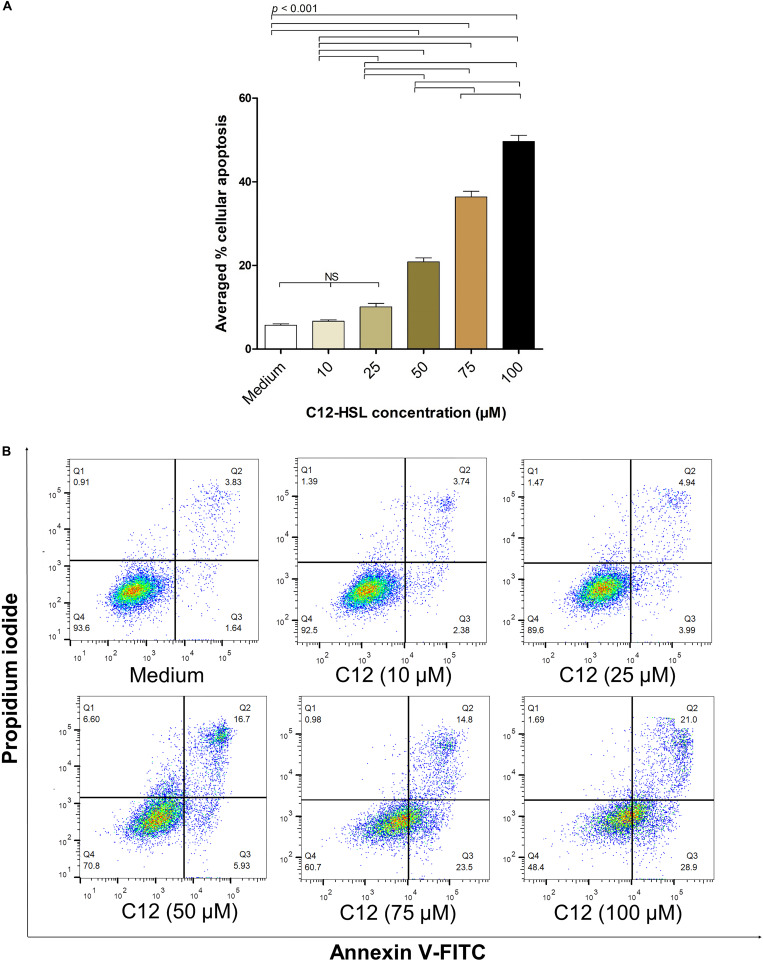
The percentages of apoptotic HeLa cells exposed to different concentrations of 3O-C12-HSL. **(A)** Bar graph of average percentages (means ± SD) of apoptotic cells from three independent experiments after exposure to 10, 25, 50, 75, and 100 μM of C12-HSL, compared with the cells in medium alone. **(B)** The density plots of HeLa cells after treatment with 10, 25, 50, 75, and 100 μM of C12-HSL, and control cells stained by Annexin V-FITC/PI and subjected to flow cytometric analysis (representative of three independent experiments). The cytotoxic activity of the 3O-C12-HSL was dose-dependent. Q1, necrotic cells (Annexin V negative/PI positive); Q2, late apoptotic cells (Annexin V positive/PI positive); Q3, early-apoptotic cells (Annexin V positive/PI negative), and Q4, viable cells (Annexin V negative/PI negative).

The average percentages of apoptotic HeLa cells treated with 10, 25, 50, 75, and 100 μM of 3O-C12-HSL dissolved in 0.25% DMSO, from three independent experiments, were 6.67 ± 0.53, 10.08 ± 1.41, 20.85 ± 1.62, 36.37 ± 2.32, and 49.63 ± 2.51%, respectively, while the background apoptotic cells of the control (cells in culture medium) was 5.71 ± 0.59% (Figure 4A). Figure 4B shows the results of the flow cytometric analysis of apoptotic cells (stained with Annexin V/PI) from one representative experiment. The background apoptotic cells (% cell viability) with and without 0.25% DMSO in the culture medium were not different (Supplementary Figure S3).

**FIGURE 5:**
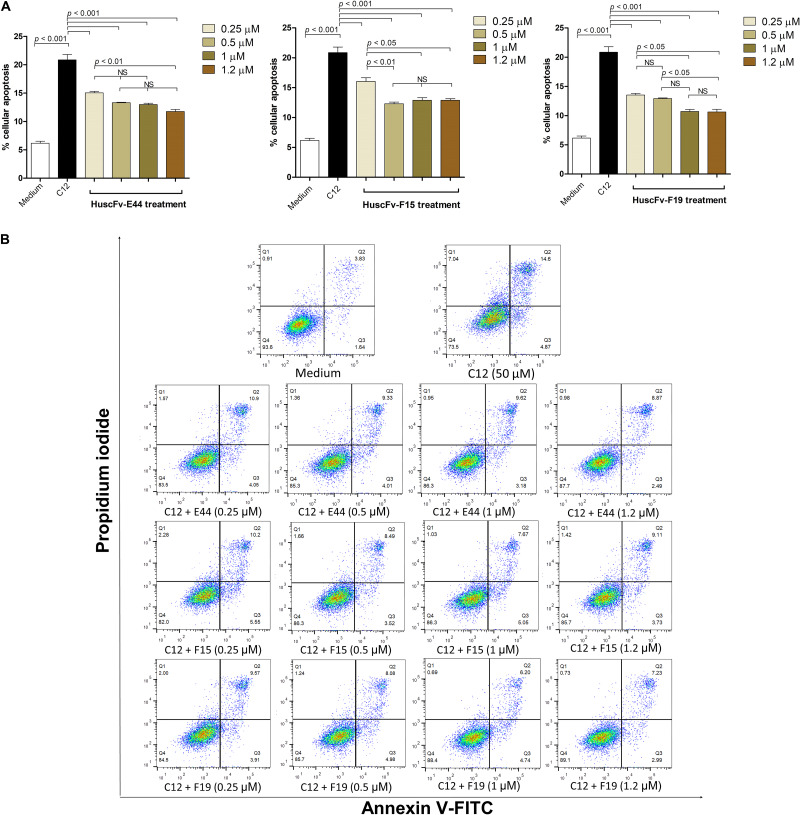
HuscFvs rescue HeLa cells from 3O-C12-HSL-mediated apoptosis. **(A)** The percentages of apoptotic cells of different treatment conditions, i.e., HeLa cells in medium alone, cells exposed to 50 μM 3O-C12-HSL (C12), cells added with 3O-C12-HSL mixed with various amounts of HuscFvs. **(B)** Density plots of flow cytometric analysis of doubly stained HeLa cells as in **(A)** (representative of one of the three reproducible experiments). The percent apoptotic cells caused by the 3O-C12-HSL was reduced significantly in the presence of HuscFvs.

The percentages of apoptotic HeLa cells exposed to 50 μM of HuscFv-bound 3O-C12-HSL (0.25, 0.5, 1.0, and 1.2 μM of individual HuscFvs) were significantly decreased compared with those without HuscFvs (Table 2 and Figure 5). The HuscFvs of all three *E. coli* clones could neutralize 3O-C12-HSL, leading to reduced HeLa-cell apoptosis.

**TABLE 2:** Flow cytometric results evaluating the efficacy of HuscFvs using Annexin V-FITC/PI staining for 3O-C12-HSL-mediated cell apoptosis.

HeLa cells treated with	Percent cellular apoptosis	Percent cellular survival
Medium alone	6.77 ± 0.20	92.50 ± 0.28
3O-C12-HSL (50 μM)	21.05 ± 2.23	72.15 ± 1.91
3O-C12-HSL + HuscFv-E44 (0.25 μM)	15.03 ± 0.49	83.57 ± 0.50
3O-C12-HSL + HuscFv-E44 (0.5 μM)	13.31 ± 0.12	85.33 ± 0.25
3O-C12-HSL + HuscFv-E44 (1.0 μM)	12.99 ± 0.41	86.10 ± 0.35
3O-C12-HSL + HuscFv-E44 (1.2 μM)	11.74 ± 0.66	87.30 ± 0.70
3O-C12-HSL + HuscFv-F15 (0.25 μM)	16.04 ± 1.06	82.07 ± 1.20
3O-C12-HSL + HuscFv-F15 (0.5 μM)	12.31 ± 0.49	85.97 ± 0.50
3O-C12-HSL + HuscFv-F15 (1.0 μM)	12.88 ± 0.75	86.27 ± 0.75
3O-C12-HSL + HuscFv-F15 (1.2 μM)	12.88 ± 0.48	85.53 ± 0.47
3O-C12-HSL + HuscFv-F19 (0.25 μM)	13.53 ± 0.46	84.27 ± 0.49
3O-C12-HSL + HuscFv-F19 (0.5 μM)	12.93 ± 0.18	85.80 ± 0.17
3O-C12-HSL + HuscFv-F19 (1.0 μM)	10.71 ± 0.60	88.67 ± 0.64
3O-C12-HSL + HuscFv-F19 (1.2 μM)	10.63 ± 0.91	88.60 ± 0.87

### HuscFv-bound-C12-HSL Had Reduced Ability to Induce sub-G1 Arrest of HeLa Cells

**FIGURE 6:**
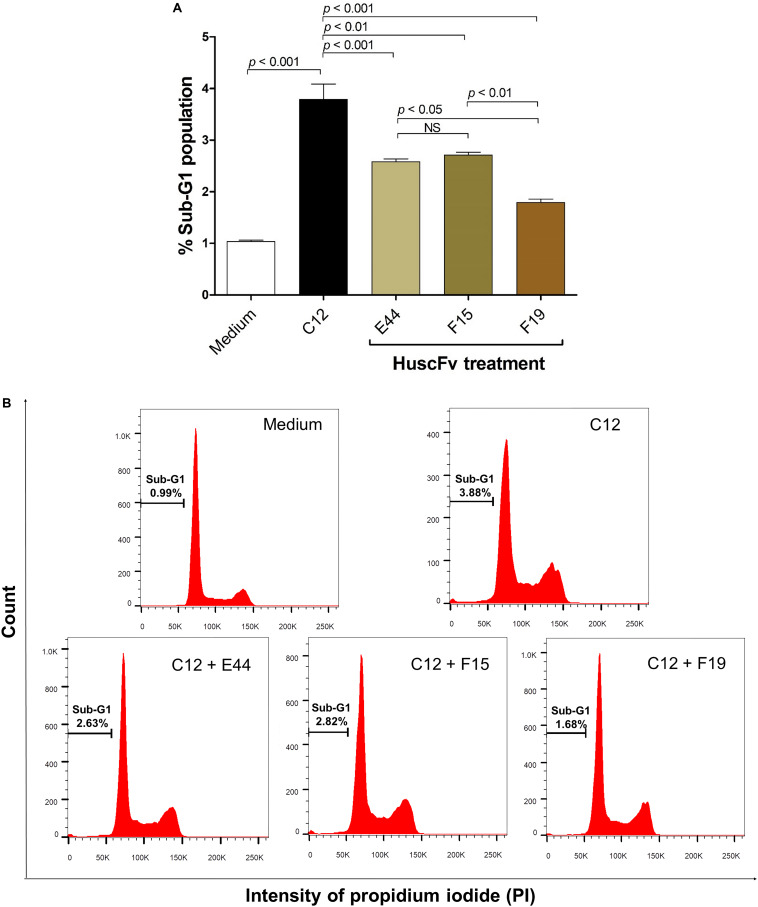
Reduction of sub-G1 arrest population of 3O-C12-HSL-exposed cells by HuscFvs. **(A)** Percent sub-G1 population (mean ± SD of triplicate individual experiments) after different treatments. **(B)** Histograms of cell cycle distribution. The percentage of apoptotic cells in the hypodiploid DNA peak (sub-G1 population) of each treatment is indicated in each plot.

Exposure of HeLa cells with 50 μM 3O-C12-HSL for 18 h resulted in 3.79 ± 0.52% of apoptotic cells in the hypodiploid DNA peak (sub-G1 population, which were apoptotic cells) as determined by flow cytometric analysis of the PI-stained cellular DNA. The numbers of cells with a hypodiploid DNA peak induced by the 3O-C12-HSL bound by the HuscFv-E44, HuscFv-F15, and HuscFv-F19, were decreased to 2.58 ± 0.10, 2.71 ± 0.10, and 1.79 ± 0.11%, respectively. The cells in medium alone had 1.04 ± 0.04% apoptotic cells (Figure 6). The results of the sub-G0/G1 analysis were conformed to those of the Annexin V/PI binding assay data.

### Degrees of Nuclear Damage Mediated by HuscFv-bound 3O-C12-HSL

**FIGURE 7:**
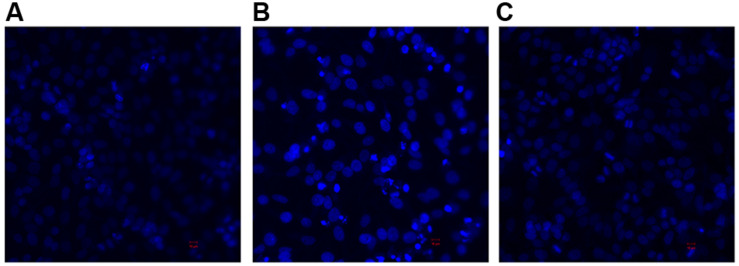
Appearances of DAPI stained-nuclei of HeLa cells after treatment with **(A)** Medium alone **(B)** 3O-C12-HSL (50 μM) and **(C)** mixture of 3O-C12-HSL (50 μM) and HuscFv-F19 (2 μM) for 18 h (original magnification 200×).

DAPI staining and confocal microscopy were used to observe the intact HeLa nuclei and nuclear DNA damage induced by the 3O-C12-HSL and the HuscFv-bound 3O-C12-HSL (Figure 7). Intact nuclei of normal HeLa cells were stained weakly by the dye (Figure 7A), while the fragmented nuclei of the 3O-C12-HSL-exposed cells were stained brightly (Figure 7B). Damage to the nuclear DNA was reduced in cells exposed to HuscFv-F19-bound 3O-C12-HSL, as shown by the dimly stained nuclei (Figure 7C).

### Mitigation of the 3O-C12-HSL Induced-mitochondrial Injuries by HuscFvs

**FIGURE 8:**
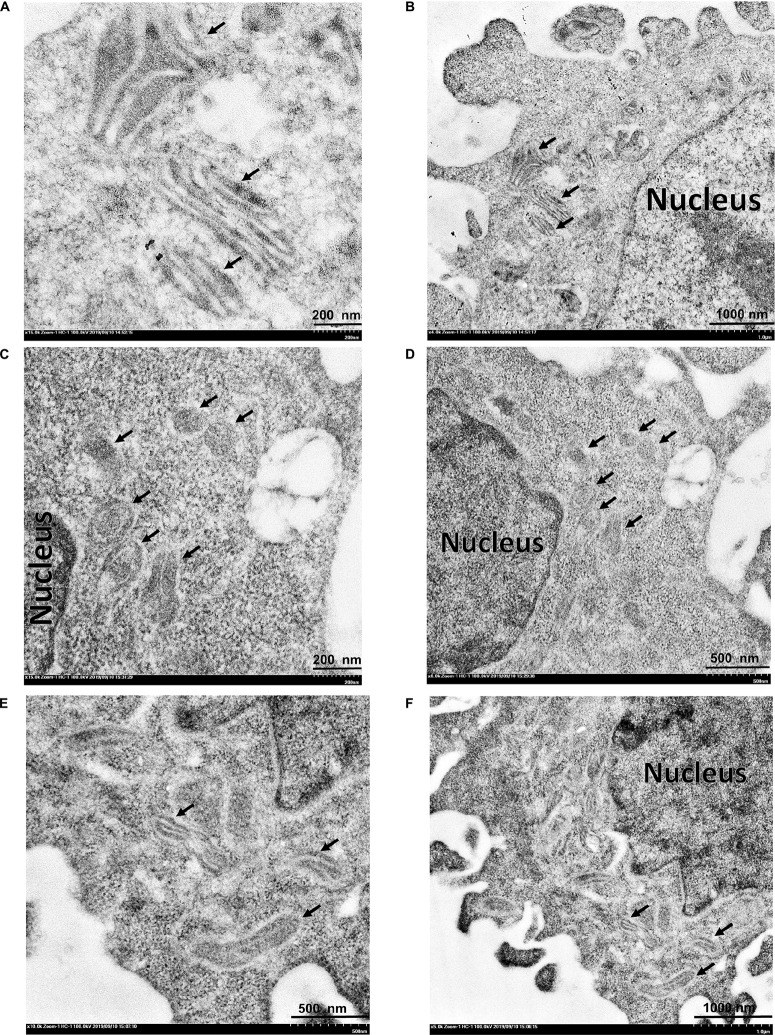
Transmission electron micrographs of mitochondrial ultrastructure. **(A,B)** normal mitochondria of HeLa cells. **(C,D)** HeLa cells treated with 3O-C12-HSL (50 μM). **(E,F)** HeLa cells treated with a mixture of 50 μM 3O-C12-HSL and 2 μM HuscFv-F19. Black arrows indicate mitochondria of the HeLa cells. Mitochondria of the cells exposed to the 3O-C12-HSL were swollen, characterized by a size increment with decreased electron density of the crista-free matrix.

Transmission electron microscopy was used to study mitochondrial changes of the HeLa cells after exposure to the 3O-C12-HSL and HuscFv-bound 3O-C12-HSL, using the cells in medium alone as a normal control. As shown in Figures 8A,B, the mitochondria of the normal cells revealed an intact mitochondrial subcellular structure. In contrast, mitochondria of the cells treated with 50 μM of 3O-C12-HSL for 18 h exhibited a swollen appearance, with single or multiple distensions of the intercellular matrix in association with severe loss of cristae and double membranes (Figures 8C,D). The pathological changes of the mitochondria were ameliorated in the cells exposed to HuscFv-F19-bound 3O-C12-HSL (representative), i.e., mild mitochondrial swelling and more cristae (Figures 8E,F), compared with the 3O-C12-HSL-exposed cells.

## Discussion

*Pseudomonas aeruginosa* 3O-C12-HSL not only regulates virulence factors of the bacteria, but also causes inflammation in the infecting host by the induction of pro-inflammatory cytokine and chemokine synthesis (Smith et al., 2002). The 3O-C12-HSL killed mammalian cells through programmed cell death, i.e., an apoptotic mechanism at concentrations ranging from 10 to 100 μM by rapidly triggering depolarization of mitochondrial membrane potential and release of cytochrome *c* into cytosol, which activates the caspase cascades (Sultan and Sokolove, 2001; Tateda et al., 2003; Kravchenko et al., 2006; Schwarzer et al., 2012; Tao et al., 2016, 2018). The apoptotic cells manifest mitochondrial permeability transition (MPT), caspase activation, nuclear fragmentation, phosphatidylserine externalization, and cell shrinkage with apoptotic bodies (Wyllie et al., 1980; Cummings and Schnellmann, 2004). Mitochondrial swelling, depolarization, and membrane permeability are the key markers of the MPT that indicates mitochondria-stimulated programmed cell death in the pathogenesis of several diseases. Upon response to external stimuli or oxidative stress, the cells undergo continuous opening of permeability transition pores (PTP) in the mitochondrial inner membrane, which augments colloidal osmotic pressure in the matrix together with mitochondrial membrane depolarization, resulting in mitochondrial swelling (Chapa-Dubocq et al., 2018) followed by rupture of the mitochondrial outer membrane and release of cytochrome *c* into the cytosol and activation of caspase cascades (Petronilli et al., 2001). The stimulated caspase-3 activates endogenous endonuclease, which cleaves nuclear DNA (Zhang and Ming, 2000). Cells with apoptotic fragmented DNA or sub-G1 population are used as a marker of apoptosis (Riccardi and Nicoletti, 2006). In this study, 3O-C12-HSL produced a significant dose-dependent increment in mammalian cell death by inducing apoptosis, which validates previous notions on the cytotoxicity of *P. aeruginosa* QS substance.

Deletion of *lasI* or *lasI* and *rhlI* diminished the lung-colonization ability of *P. aeruginosa* in a mouse model of acute pneumonitis (Smith et al., 2002). *P. aeruginosa* mutants with defective QS are known to have less virulence and be more susceptible to antibiotic treatments and host immunity than the respective wild-type (Hentzer et al., 2003). As such, *P. aeruginosa* QS systems are attractive targets for direct-acting therapeutic agents, of which the expected treatment consequences are mitigation of the severity of the bacteria-associated diseases (Penesyan et al., 2015). During the past decades, several groups of *P. aeruginosa* QS inhibitors/modulators have been identified: small chemical molecules, i.e., AHL analogs (phenylpropionyl homoserine lactones and phenyloxyacetyl homoserine lactones of the *N*-aryl homoserine lactone library) (Geske et al., 2008), *N*-acyl cyclopentylamides (Ishida et al., 2007), halogenated furanone compound (Hentzer et al., 2002), other furanone derivatives (Kim et al., 2008), aspirin (El-Mowafy et al., 2014), and itaconimides and citraconimides (Fong et al., 2018); and natural inhibitors, such as secondary metabolites of the Australian marine macroalgae, *Delisea pulchra* (Givskov et al., 1996), patulin and penicillic acid from extracts of *Penicillium* species (Rasmussen et al., 2005), an organosulfur compound found in garlic extracts, named Ajoene (Jakobsen et al., 2012), and derivatives of ellagic acid (dilactone of hexahydroxydiphenic acid) from black or chebulic myrobalan, *Terminalia chebula* Retz (Sarabhai et al., 2013). Unfortunately, these compounds are relatively toxic to mammalian cells, which limits their therapeutic use (Ni et al., 2009). Recently, natural plant-derived compounds, *trans*-cinnamaldehyde (CA), and salicylic acid (SA) have been shown to effectively downregulate both *las* and *rhl* QS systems, reduce the production of extracellular virulence factors, i.e., protease, elastase, and pyocyanin, and reduce biofilm formation, concomitantly with repressed rhamnolipid gene expression (Ahmed et al., 2019). However, the sole use of QS inhibitors at high concentrations to eradicate bacterial infection completely is of legitimate concern due to potential toxicity (Shreaz et al., 2016).

Passive immunization has been used as an intervention for post-exposure morbidity and/or treatment of diseases since the late 18th century (Keller and Stiehm, 2000). An antibody molecule uses multiple amino acid residues in several CDRs (sometimes with the help of FRs) to form multiple non-covalent bonds with the target molecule, thus, making it difficult for pathogens to create antibody escape mutants, compared with small molecular drugs/inhibitors, from which resistant variants emerge rather easily and frequently. Therapeutic antibodies may be in the form of intact molecules (two antigen-binding sites with Fc fragment- when the bioactivities of the Fc are required for effectiveness) or merely smaller antibody fragments, i.e., F(ab′)_2_, Fab, scFv, or single domain (VH, V_H_H) with higher tissue penetrating ability than the intact four-chain counterpart when the Fc is dispensable. For *P. aeruginosa* infection, specific murine mAb, RS2-1G9, directed toward bacterial 3O-C12-HSL has been generated for use as an immunotherapeutic agent (Kaufmann et al., 2006, 2008). This murine antibody displayed the cytoprotective effect of 3O-C12-HSL-exposed host cells (Kaufmann et al., 2006, 2008; Debler et al., 2007). In addition, sheep-mouse chimeric mAb recognized native AHL protected mice from lethal *P. aeruginosa* infection (Palliyil et al., 2014). Nevertheless, while these 3O-C12-HSL-specific antibodies have therapeutic potential, their immunogenicity in human recipients, with possible adverse consequences, such as serum sickness, should be of concern.

Nowadays, any engineered fully human antibody format can be generated *in vitro* using phage display technology, invented by Nobel laureate, George Pearson Smith (Smith, 1985) as a biological tool (Santajit et al., 2019). The target antigens, such as proteins or peptides, attached to a carrier surface, e.g., fixed cell, plastic bead, or well of an ELISA plate, can be used as bait to fish out phage clones that display recombinant antibodies binding to the antigen from an antibody phage display library (Kulkeaw et al., 2009). Suppressor *E. coli*, such as strain HB2151 transfected with antigen-bound phages, when grown in appropriate conditioned medium, produces antigen-specific antibodies, and these antibodies can be isolated from the bacterial lysate/homogenate (Glab-Ampai et al., 2017; Santajit et al., 2019). Nevertheless, attachment of the small molecular haptens, like 3O-C12-HSL, to solid surfaces (as well as retaining the native configuration of the molecule) for conventional phage biopanning, is a relatively complicated process compared with proteins or peptides. Therefore, in this study, an alternative method was used to produce fully human scFvs (HuscFvs) to the synthetic *P. aeruginosa* 3O-C12-HSL based on the principle of antibody polyspecificity and antigenic molecular mimicry, i.e., completely unrelated molecules can share common receptors, possibly through similar structural and/or chemical features involved in recognition and binding (Wing, 1995; Tapryal et al., 2013). A repertoire of *E. coli* clones carrying recombinant *huscfv*-phagemids was previously retrieved from a HuscFv phage display library (Kulkeaw et al., 2009) by panning with *P. aeruginosa* exotoxin A (Santajit et al., 2019). Moreover, because the previously produced murine mAb, RS2-1G9, has been known as the *P. aeruginosa* quorum quencher, we used computerized antibody structure superimposition to select the bacterial derived-HuscFvs that shared structural homology with the murine mAb RS2-1G9 antigen-binding site. Using this method, the HuscFvs of three phagemid-transformed *E. coli* clones (E44, F15, and F19) showed high and satisfactory degrees of molecular similarity to the mAb RS2-1G9 antigen-binding site. Besides, these HuscFvs could neutralize the cytotoxic effects of the 3O-C12-HSL in the induction of cellular apoptosis. The HuscFv bound-3O-C12-HSL had a reduced capacity to mediate mitochondrial swelling, diminishing DNA damage and reducing sub-G1 arrest population of exposed cells. Unfortunately, the amount of C12-HSL inside the HeLa cells with and without HuscFv treatments were not measured; therefore, it is not known whether the HuscFvs could prevent HSL from entering the cells. Although the actual 3O-C12-HSL neutralizing mechanism of the HuscFvs needs laboratory investigation, the predicted structural complexes between the QS (ligand) and the HuScFvs (receptors) indicated that the latter used several residues in different CDRs and FRs to interact non-covalently with the target, including van der Waals’ forces, hydrophobic interactions, and hydrogen bonds. These interactions might render the disarming of the bacterial toxic molecule through C12-HSL signal interference, which would mitigate bacterial disease severity. This perspective needs further testing of the HuscFvs on *P. aeruginosa* phenotypes both *in vitro* (bacterial culture), such as expression of the QS controlled virulence factors, as well as in the *in vivo* model of bacterial infection.

## Conclusion

The engineered human single-chain variable fragments that attenuated the potent cytotoxicity of the *P. aeruginosa* quorum sensing molecule, 3O-C12-HSL, were generated successfully through the molecular basis of antibody polyspecificity and antigenic mimicry. The fully human antibody fragments rescued mammalian cells from the 3O-C12-HSL-mediated mitochondrial injuries, DNA damage, and cellular apoptosis *in vitro*. They should be tested further by step-by-step *in vivo* toward the clinical application as a sole or an adjunct therapy for the failing antibiotic treatment of *P. aeruginosa* infections.

## Data Availability Statement

All datasets generated for this study are included in the article/Supplementary Material.

## Author Contributions

NI and WC conceived the project, analyzed the data, and edited the manuscript. SS did most of the experiments, drafted the manuscript, and prepared the figures. KM helped SS with flow cytometric analysis. WS advised SS on recombinant HuscFv production. PP supervised SS on fluorescence staining and confocal microscopy. SA performed the electron microscopy. NS, OR, and MC-N helped NI and WC to analyze the data and made comments. All authors critically reviewed the manuscript and gave final approval for publication.

## Conflict of Interest

The authors declare that the research was conducted in the absence of any commercial or financial relationships that could be construed as a potential conflict of interest.

## References

[B1] Abhinandan K. R. Martin A. C. (2008). Analysis and improvements to Kabat and structurally correct numbering of antibody variable domains. *Mol. Immunol.* 45 3832–3839. 10.1016/j.molimm.2008.05.022 18614234

[B2] Ahmed S. A. K. S. Rudden M. Smyth T. J. Dooley J. S. Marchant R. Banat I. M. (2019). Natural quorum sensing inhibitors effectively downregulate gene expression of *Pseudomonas aeruginosa* virulence factors. *Appl. Microbiol. Biotechnol.* 103 3521–3535. 10.1007/s00253-019-09618-0 30852658 PMC6449319

[B3] Bleves S. Soscia C. Nogueira-Orlandi P. Lazdunski A. Filloux A. (2005). Quorum sensing negatively controls type III secretion regulon expression in *Pseudomonas aeruginosa* PAO1. *J. Bacteriol.* 187 3898–3902. 10.1128/jb.187.11.3898-3902.2005 15901720 PMC1112058

[B4] Boontham P. Robins A. Chandran P. Pritchard D. Cámara M. Williams P. (2008). Significant immunomodulatory effects of *Pseudomonas aeruginosa* quorum-sensing signal molecules: possible link in human sepsis. *Clin. Sci.* 115 343–351. 10.1042/cs20080018 18363571

[B5] Chapa-Dubocq X. Makarov V. Javadov S. (2018). Simple kinetic model of mitochondrial swelling in cardiac cells. *J. Cell. Physiol.* 233 5310–5321. 10.1002/jcp.26335 29215716 PMC6063534

[B6] Cummings B. S. Schnellmann R. G. (2004). Measurement of cell death in mammalian cells. *Curr. Protoc. Pharmacol.* 25 12–18. 10.1002/0471141755.ph1208s25 PMC3874588 22294120

[B7] Debler E. W. Kaufmann G. F. Kirchdoerfer R. N. Mee J. M. Janda K. D. Wilson I. A. (2007). Crystal structures of a quorum-quenching antibody. *J. Mol. Biol.* 368 1392–1402. 10.1016/j.jmb.2007.02.081 17400249 PMC1994716

[B8] Defoirdt T. Boon N. Bossier P. (2010). Can bacteria evolve resistance to quorum sensing disruption? *PLoS Pathog.* 6:e1000989. 10.1371/journal.ppat.1000989 20628566 PMC2900297

[B9] Duan K. Surette M. G. (2007). Environmental regulation of *Pseudomonas aeruginosa* PAO1 Las and Rhl quorum-sensing systems. *J. Bacteriol.* 189 4827–4836. 10.1128/jb.00043-07 17449617 PMC1913434

[B10] Eberhard A. Burlingame A. L. Eberhard C. Kenyon G. L. Nealson K. H. Oppenheimer N. J. (1981). Structural identification of autoinducer of *Photobacterium fischeri* luciferase. *Biochemistry* 20 2444–2449. 10.1021/bi00512a013 7236614

[B11] El-Mowafy S. A. El Galil K. H. A. El-Messery S. M. Shaaban M. I. (2014). Aspirin is an efficient inhibitor of quorum sensing, virulence and toxins in *Pseudomonas aeruginosa*. *Microb. Pathog.* 74 25–32. 10.1016/j.micpath.2014.07.008 25088031

[B12] Fong J. Mortensen K. T. Nørskov A. Qvortrup K. Yang L. Tan C. H. (2018). Itaconimides as novel quorum sensing inhibitors of *Pseudomonas aeruginosa*. *Front. Cell. Infect. Microbiol.* 8:443. 10.3389/fcimb.2018.00443 30666301 PMC6330316

[B13] Forli S. Huey R. Pique M. E. Sanner M. F. Goodsell D. S. Olson A. J. (2016). Computational protein–ligand docking and virtual drug screening with the AutoDock suite. *Nat. Protoc.* 11 905–919. 10.1038/nprot.2016.051 27077332 PMC4868550

[B14] Geske G. D. Mattmann M. E. Blackwell H. E. (2008). Evaluation of a focused library of N-aryl L-homoserine lactones reveals a new set of potent quorum sensing modulators. *Bioorg. Med. Chem. Lett.* 18 5978–5981. 10.1016/j.bmcl.2008.07.089 18760602 PMC2593151

[B15] Givskov M. de Nys R. Manefield M. Gram L. Maximilien R. I. A. Eberl L. E. O. (1996). Eukaryotic interference with homoserine lactone-mediated prokaryotic signalling. *J. Bacteriol.* 178 6618–6622. 10.1128/jb.178.22.6618-6622.1996 8932319 PMC178549

[B16] Glab-Ampai K. Chulanetra M. Malik A. A. Juntadech T. Thanongsaksrikul J. Srimanote P. (2017). Human single chain-transbodies that bound to domain-I of non-structural protein 5A (NS5A) of hepatitis C virus. *Sci. Rep.* 7:15042. 10.1038/s41598-017-14886-9 PMC5678119 29118372

[B17] Hentzer M. Riedel K. Rasmussen T. B. Heydorn A. Andersen J. B. Parsek M. R. (2002). Inhibition of quorum sensing in *Pseudomonas aeruginosa* biofilm bacteria by a halogenated furanone compound. *Microbiology* 148 87–102. 10.1099/00221287-148-1-87 11782502

[B18] Hentzer M. Wu H. Andersen J. B. Riedel K. Rasmussen T. B. Bagge N. (2003). Attenuation of *Pseudomonas aeruginosa* virulence by quorum sensing inhibitors. *EMBO J.* 22 3803–3815. 10.1093/emboj/cdg366 12881415 PMC169039

[B19] Ishida T. Ikeda T. Takiguchi N. Kuroda A. Ohtake H. Kato J. (2007). Inhibition of quorum sensing in *Pseudomonas aeruginosa* by N-acyl cyclopentylamides. *Appl. Environ. Microbiol.* 73 3183–3188. 10.1128/aem.02233-06 17369333 PMC1907104

[B20] Jacobi C. A. Schiffner F. Henkel M. Waibel M. Stork B. Daubrawa M. (2009). Effects of bacterial N-acyl homoserine lactones on human Jurkat T lymphocytes-OdDHL induces apoptosis via the mitochondrial pathway. *Int. J. Med. Microbiol.* 299 509–519. 10.1016/j.ijmm.2009.03.005 19464950

[B21] Jakobsen T. H. van Gennip M. Phipps R. K. Shanmugham M. S. Christensen L. D. Alhede M. (2012). Ajoene, a sulfur-rich molecule from garlic, inhibits genes controlled by quorum sensing. *Antimicrob. Agents Chemother.* 56 2314–2325. 10.1128/aac.05919-11 22314537 PMC3346669

[B22] Jittavisutthikul S. Seesuay W. Thanongsaksrikul J. Thueng-in K. Srimanote P. Werner R. G. (2016). Human transbodies to HCV NS3/4A protease inhibit viral replication and restore host innate immunity. *Front. Immunol.* 7 318. 10.3389/fimmu.2016.00318 27617013 PMC4999588

[B23] Kalia V. C. Wood T. K. Kumar P. (2014). Evolution of resistance to quorum-sensing inhibitors. *Microb. Ecol.* 68 13–23. 24194099 10.1007/s00248-013-0316-y PMC4012018

[B24] Kaufmann G. F. Park J. Mee J. M. Ulevitch R. J. Janda K. D. (2008). The quorum quenching antibody RS2-1G9 protects macrophages from the cytotoxic effects of the *Pseudomonas aeruginosa* quorum sensing signaling molecule N-3-oxo-dodecanoyl-homoserine lactone. *Mol. Immunol.* 45 2710–2714. 10.1016/j.molimm.2008.01.010 18304641 PMC2359578

[B25] Kaufmann G. F. Sartorio R. Lee S. H. Mee J. M. Altobell L. J. Kujawa D. P. (2006). Antibody interference with N-acyl homoserine lactone-mediated bacterial quorum sensing. *J. Am. Chem. Soc.* 128 2802–2803. 10.1021/ja0578698 16506750 PMC2546487

[B26] Keller M. A. Stiehm E. R. (2000). Passive immunity in prevention and treatment of infectious diseases. *Clin. Microbiol. Rev.* 13 602–614. 10.1128/cmr.13.4.602 11023960 PMC88952

[B27] Kim C. Kim J. Park H. Y. Park H. J. Lee J. H. Kim C. K. (2008). Furanone derivatives as quorum-sensing antagonists of *Pseudomonas aeruginosa*. *Appl. Microbiol. Biotechnol.* 80 37–47. 18566810 10.1007/s00253-008-1474-6

[B28] Kim S. Thiessen P. A. Bolton E. E. Chen J. Fu G. Gindulyte A. (2015). PubChem substance and compound databases. *Nucleic Acids Res.* 44 D1202–D1213. 26400175 10.1093/nar/gkv951 PMC4702940

[B29] Kravchenko V. V. Kaufmann G. F. Mathison J. C. Scott D. A. Katz A. Z. Wood M. R. (2006). N-(3-oxo-acyl) homoserine lactones signal cell activation through a mechanism distinct from the canonical pathogen-associated molecular pattern recognition receptor pathways. *J. Biol. Chem.* 281 28822–28830. 10.1074/jbc.m606613200 16893899

[B30] Krzyżek P. (2019). Challenges and limitations of anti-quorum sensing therapies. *Front. Microbiol.* 10:2473. 10.3389/fmicb.2019.02473 31736912 PMC6834643

[B31] Kulkeaw K. Sakolvaree Y. Srimanote P. Tongtawe P. Maneewatch S. Sookrung N. (2009). Human monoclonal ScFv neutralize lethal Thai cobra. *Naja kaouthia*, neurotoxin. *J. Proteomics* 72 270–280. 19162253 10.1016/j.jprot.2008.12.007

[B32] Laskowski R. A. MacArthur M. W. Moss D. S. Thornton J. M. (1993). PROCHECK: a program to check the stereochemical quality of protein structures. *J. Appl. Crystallogr.* 26 283–291. 10.1107/s0021889892009944

[B33] Li L. Hooi D. Chhabra S. R. Pritchard D. Shaw P. E. (2004). Bacterial N-acylhomoserine lactone-induced apoptosis in breast carcinoma cells correlated with down-modulation of STAT3. *Oncogene* 23 4894–4902. 10.1038/sj.onc.1207612 15064716

[B34] Liu Y. C. Chan K. G. Chang C. Y. (2015). Modulation of host biology by *Pseudomonas aeruginosa* quorum sensing signal molecules: messengers or traitors. *Front. Microbiol.* 6:1226. 10.3389/fmicb.2015.01226 26617576 PMC4637427

[B35] López-Jácome L. E. Garza Ramos-Martínez G. Hernández-Durán M. Franco-Cendejas R. Loarca D. Romero-Martínez D. (2019). AiiM lactonase strongly reduces quorum sensing controlled virulence factors in clinical strains of *Pseudomonas aeruginosa* isolated from burned patients. *Front. Microbiol.* 10:2657. 10.3389/fmicb.2019.02657 31798568 PMC6868103

[B36] Maeda T. García-Contreras R. Pu M. Sheng L. Garcia L. R. Tomás M. (2012). Quorum quenching quandary: resistance to antivirulence compounds. *ISME J.* 6 493–501. 10.1038/ismej.2011.122 21918575 PMC3280137

[B37] Malhotra S. Hayes D. Wozniak D. J. (2019). Cystic fibrosis and *Pseudomonas aeruginosa*: the host-microbe interface. *Clin. Microbiol. Rev.* 32:e00138-18. 10.1128/CMR.00138-18 PMC6589863 31142499

[B38] Miyairi S. Tateda K. Fuse E. T. Ueda C. Saito H. Takabatake T. (2006). Immunization with 3-oxododecanoyl-L-homoserine lactone-protein conjugate protects mice from lethal *Pseudomonas aeruginosa* lung infection. *J. Med. Microbiol.* 55 1381–1387. 10.1099/jmm.0.46658-0 17005787

[B39] Moradali M. F. Ghods S. Rehm B. H. (2017). *Pseudomonas aeruginosa* lifestyle: a paradigm for adaptation, survival, and persistence. *Front. Cell. Infect. Microbiol.* 7:39 10.3389/fcimb.2017.00039 PMC5310132 28261568

[B40] Nguyen M. N. Tan K. P. Madhusudhan M. S. (2011). CLICK—topology-independent comparison of biomolecular 3D structures. *Nucleic Acids Res.* 39 W24–W28. 21602266 10.1093/nar/gkr393 PMC3125785

[B41] Ni N. Li M. Wang J. Wang B. (2009). Inhibitors and antagonists of bacterial quorum sensing. *Med. Res. Rev.* 29 65–124. 10.1002/med.20145 18956421

[B42] O’Connor G. Knecht L. D. Salgado N. Strobel S. Pasini P. Daunert S. (2015). Whole-cell biosensors as tools for the detection of quorum-sensing molecules: uses in diagnostics and the investigation of the quorum-sensing mechanism. *Adv. Biochem. Eng. Biotechnol.* 154 181–200. 10.1007/10_2015_337 26475469

[B43] Palliyil S. Broadbent I. D. (2009). Novel immunotherapeutic approaches to the treatment of infections caused by Gram-negative bacteria. *Curr. Opin. Pharmacol.* 9 566–570. 10.1016/j.coph.2009.07.007 19726227

[B44] Palliyil S. Downham C. Broadbent I. Charlton K. Porter A. J. (2014). High-sensitivity monoclonal antibodies specific for homoserine lactones protect mice from lethal *Pseudomonas aeruginosa* infections. *Appl. Environ. Microbiol.* 80 462–469. 10.1128/aem.02912-13 24185854 PMC3911118

[B45] Pearson J. P. Gray K. M. Passador L. Tucker K. D. Eberhard A. Iglewski B. H. (1994). Structure of the autoinducer required for expression of *Pseudomonas aeruginosa* virulence genes. *Proc. Natl. Acad. Sci. U.S.A.* 91 197–201. 10.1073/pnas.91.1.197 8278364 PMC42913

[B46] Pearson J. P. Passador L. Iglewski B. H. Greenberg E. P. (1995). A second N-acylhomoserine lactone signal produced by *Pseudomonas aeruginosa*. *Proc. Natl. Acad. Sci. U.S.A.* 92 1490–1494. 10.1073/pnas.92.5.1490 7878006 PMC42545

[B47] Penesyan A. Gillings M. Paulsen I. T. (2015). Antibiotic discovery: combatting bacterial resistance in cells and in biofilm communities. *Molecules* 20 5286–5298. 10.3390/molecules20045286 25812150 PMC6272253

[B48] Petronilli V. Penzo D. Scorrano L. Bernardi P. Di Lisa F. (2001). The mitochondrial permeability transition, release of cytochrome *c* and cell death correlation with the duration of pore openings *in situ*. *J. Biol. Chem.* 276 12030–12034. 10.1074/jbc.m010604200 11134038

[B49] Preston M. J. Seed P. C. Toder D. S. Iglewski B. H. Ohman D. E. Gustin J. K. (1997). Contribution of proteases and LasR to the virulence of *Pseudomonas aeruginosa* during corneal infections. *Infect. Immun.* 65 3086–3090. 10.1128/iai.65.8.3086-3090.1997 9234758 PMC175435

[B50] Rasamiravaka T. El Jaziri M. (2016). Quorum-sensing mechanisms and bacterial response to antibiotics in *P. aeruginosa*. *Curr. Microbiol.* 73 747–753. 10.1007/s00284-016-1101-1 27449213

[B51] Rasmussen T. B. Givskov M. (2006). Quorum-sensing inhibitors as anti-pathogenic drugs. *Int. J. Med. Microbiol.* 296 149–161. 10.1016/j.ijmm.2006.02.005 16503194

[B52] Rasmussen T. B. Skindersoe M. E. Bjarnsholt T. Phipps R. K. Christensen K. B. Jensen P. O. (2005). Identity and effects of quorum-sensing inhibitors produced by *Penicillium* species. *Microbiology* 151 1325–1340. 10.1099/mic.0.27715-0 15870443

[B53] Riccardi C. Nicoletti I. (2006). Analysis of apoptosis by propidium iodide staining and flow cytometry. *Nat. Protoc.* 1 1458–1461. 10.1038/nprot.2006.238 17406435

[B54] Ritchie A. J. Whittall C. Lazenby J. J. Chhabra S. R. Pritchard D. I. Cooley M. A. (2007). The immunomodulatory *Pseudomonas aeruginosa* signalling molecule N-(3-oxododecanoyl)-l-homoserine lactone enters mammalian cells in an unregulated fashion. *Immunol. Cell. Biol.* 85 596–602. 10.1038/sj.icb.7100090 17607318

[B55] Rutherford S. T. Bassler B. L. (2012). Bacterial quorum sensing: its role in virulence and possibilities for its control. *Cold Spring Harb. Perspect. Med.* 2:a012427. 10.1101/cshperspect.a012427 23125205 PMC3543102

[B56] Sadikot R. T. Blackwell T. S. Christman J. W. Prince A. S. (2005). Pathogen-host interactions in *Pseudomonas aeruginosa* pneumonia. *Am. J. Respir. Crit. Care Med.* 171 1209–1223. 15695491 10.1164/rccm.200408-1044SO PMC2718459

[B57] Santajit S. Seesuay W. Mahasongkram K. Sookrung N. Ampawong S. Reamtong O. (2019). Human single-chain antibodies that neutralize *Pseudomonas aeruginosa*-exotoxin A-mediated cellular apoptosis. *Sci. Rep.* 9 1–15. 31624289 10.1038/s41598-019-51089-w PMC6797803

[B58] Sarabhai S. Sharma P. Capalash N. (2013). Ellagic acid derivatives from *Terminalia chebula* Retz. downregulate the expression of quorum sensing genes to attenuate *Pseudomonas aeruginosa* PAO1 virulence. *PLoS One* 8:e53441. 10.1371/journal.pone.0053441 23320085 PMC3539995

[B59] Schwarzer C. Fu Z. Patanwala M. Hum L. Lopez-Guzman M. Illek B. (2012). *Pseudomonas aeruginosa* biofilm-associated homoserine lactone C12 rapidly activates apoptosis in airway epithelia. *Cell. Microbiol.* 14 698–709. 10.1111/j.1462-5822.2012.01753.x 22233488 PMC4112999

[B60] Shiner E. K. Terentyev D. Bryan A. Sennoune S. Martinez-Zaguilan R. Li G. (2006). *Pseudomonas aeruginosa* autoinducer modulates host cell responses through calcium signalling. *Cell. Microbiol.* 8 1601–1610. 10.1111/j.1462-5822.2006.00734.x 16984415

[B61] Shreaz S. Wani W. A. Behbehani J. M. Raja V. Irshad M. Karched M. (2016). Cinnamaldehyde and its derivatives, a novel class of antifungal agents. *Fitoterapia* 112 116–131. 10.1016/j.fitote.2016.05.016 27259370

[B62] Silva Filho L. V. R. F. Ferreira F. D. A. Reis F. J. C. Britto M. C. A. D. Levy C. E. Clark O. (2013). *Pseudomonas aeruginosa* infection in patients with cystic fibrosis: scientific evidence regarding clinical impact, diagnosis, and treatment. *J. Bras. Pneumol.* 39 495–512. 10.1590/s1806-37132013000400015 24068273 PMC4075866

[B63] Smith G. P. (1985). Filamentous fusion phage: novel expression vectors that display cloned antigens on the virion surface. *Science* 228 1315–1317. 10.1126/science.4001944 4001944

[B64] Smith R. S. Harris S. G. Phipps R. Iglewski B. (2002). The *Pseudomonas aeruginosa* quorum-sensing molecule N-(3-oxododecanoyl) homoserine lactone contributes to virulence and induces inflammation *in vivo*. *J. Bacteriol.* 184 1132–1139. 10.1128/jb.184.4.1132-1139.2002 11807074 PMC134808

[B65] Soto-Aceves M. P. Cocotl-Yañez M. Merino E. Castillo-Juárez I. Cortés-López H. González-Pedrajo B. (2019). Inactivation of the quorum-sensing transcriptional regulators LasR or RhlR does not suppress the expression of virulence factors and the virulence of *Pseudomonas aeruginosa* PAO1. *Microbiology* 165 425–432. 10.1099/mic.0.000778 30707095

[B66] Sultan A. Sokolove P. M. (2001). Free fatty acid effects on mitochondrial permeability: an overview. *Arch. Biochem. Biophys.* 386 52–61. 10.1006/abbi.2000.2195 11361000

[B67] Tang H. B. DiMango E. Bryan R. Gambello M. Iglewski B. H. Goldberg J. B. (1996). Contribution of specific *Pseudomonas aeruginosa* virulence factors to pathogenesis of pneumonia in a neonatal mouse model of infection. *Infect. Immun.* 64 37–43. 10.1128/iai.64.1.37-43.1996 8557368 PMC173724

[B68] Tao S. Luo Y. He B. Liu J. Qian X. Ni Y. (2016). Paraoxonase 2 modulates a proapoptotic function in LS174T cells in response to quorum sensing molecule N-(3-oxododecanoyl)-L-homoserine lactone. *Sci. Rep.* 6:28778. 10.1038/srep28778 PMC4929476 27364593

[B69] Tao S. Niu L. Cai L. Geng Y. Hua C. Ni Y. (2018). N-(3-oxododecanoyl)-l-homoserine lactone modulates mitochondrial function and suppresses proliferation in intestinal goblet cells. *Life Sci.* 201 81–88. 10.1016/j.lfs.2018.03.049 29596921

[B70] Tapryal S. Gaur V. Kaur K. J. Salunke D. M. (2013). Structural evaluation of a mimicry-recognizing paratope: plasticity in antigen–antibody interactions manifests in molecular mimicry. *J. Immunol.* 191 456–463. 10.4049/jimmunol.1203260 23733869

[B71] Tateda K. Ishii Y. Horikawa M. Matsumoto T. Miyairi S. Pechere J. C. (2003). The *Pseudomonas aeruginosa* autoinducer N-3-oxododecanoyl homoserine lactone accelerates apoptosis in macrophages and neutrophils. *Infect. Immun.* 71 5785–5793. 10.1128/iai.71.10.5785-5793.2003 14500500 PMC201082

[B72] Trott O. Olson A. J. (2010). AutoDock Vina: improving the speed and accuracy of docking with a new scoring function, efficient optimization, and multithreading. *J. Comput. Chem.* 31 455–461. 19499576 10.1002/jcc.21334 PMC3041641

[B73] Venturi V. (2006). Regulation of quorum sensing in *Pseudomonas*. *FEMS Microbiol. Rev.* 30 274–291. 16472307 10.1111/j.1574-6976.2005.00012.x

[B74] Wagner V. E. Bushnell D. Passador L. Brooks A. I. Iglewski B. H. (2003). Microarray analysis of *Pseudomonas aeruginosa* quorum-sensing regulons: effects of growth phase and environment. *J. Bacteriol.* 185 2080–2095. 10.1128/jb.185.7.2080-2095.2003 12644477 PMC151498

[B75] Waters C. M. Goldberg J. B. (2019). *Pseudomonas aeruginosa* in cystic fibrosis: a chronic cheater. *Proc. Natl. Acad. Sci. U.S.A.* 116 6525–6527. 30890646 10.1073/pnas.1902734116 PMC6452658

[B76] Wing M. G. (1995). The molecular basis for a polyspecific antibody. *Clin. Exp. Immunol.* 99 313–315. 10.1111/j.1365-2249.1995.tb05551.x 7882551 PMC1534201

[B77] Wyllie A. H. Kerr J. R. Currie A. R. (1980). Cell death: the significance of apoptosis. *Int. Rev. Cytol.* 68 251–306. 10.1016/s0074-7696(08)62312-8 7014501

[B78] Xu D. Zhang Y. (2011). Improving the physical realism and structural accuracy of protein models by a two-step atomic-level energy minimization. *Biophys. J.* 101 2525–2534. 10.1016/j.bpj.2011.10.024 22098752 PMC3218324

[B79] Yang J. Yan R. Roy A. Xu D. Poisson J. Zhang Y. (2015). The I-TASSER suite: protein structure and function prediction. *Nat. Methods* 12 7–8. 10.1038/nmeth.3213 25549265 PMC4428668

[B80] Zhang J. H. Ming X. U. (2000). DNA fragmentation in apoptosis. *Cell Res.* 10 205–211. 11032172 10.1038/sj.cr.7290049

