# Supplementary material for: Human Single-chain Variable Fragments Neutralize Pseudomonas aeruginosa Quorum Sensing Molecule, 3O-C12-HSL, and Prevent Cells From the HSL-mediated Apoptosis
Source: Front Microbiol. 2020 Jun 24;11:1172. doi: 10.3389/fmicb.2020.01172 (PMC7326786; doi:10.3389/fmicb.2020.01172)
Supplement: Supplementary file 1 [file Data_Sheet_1.doc]

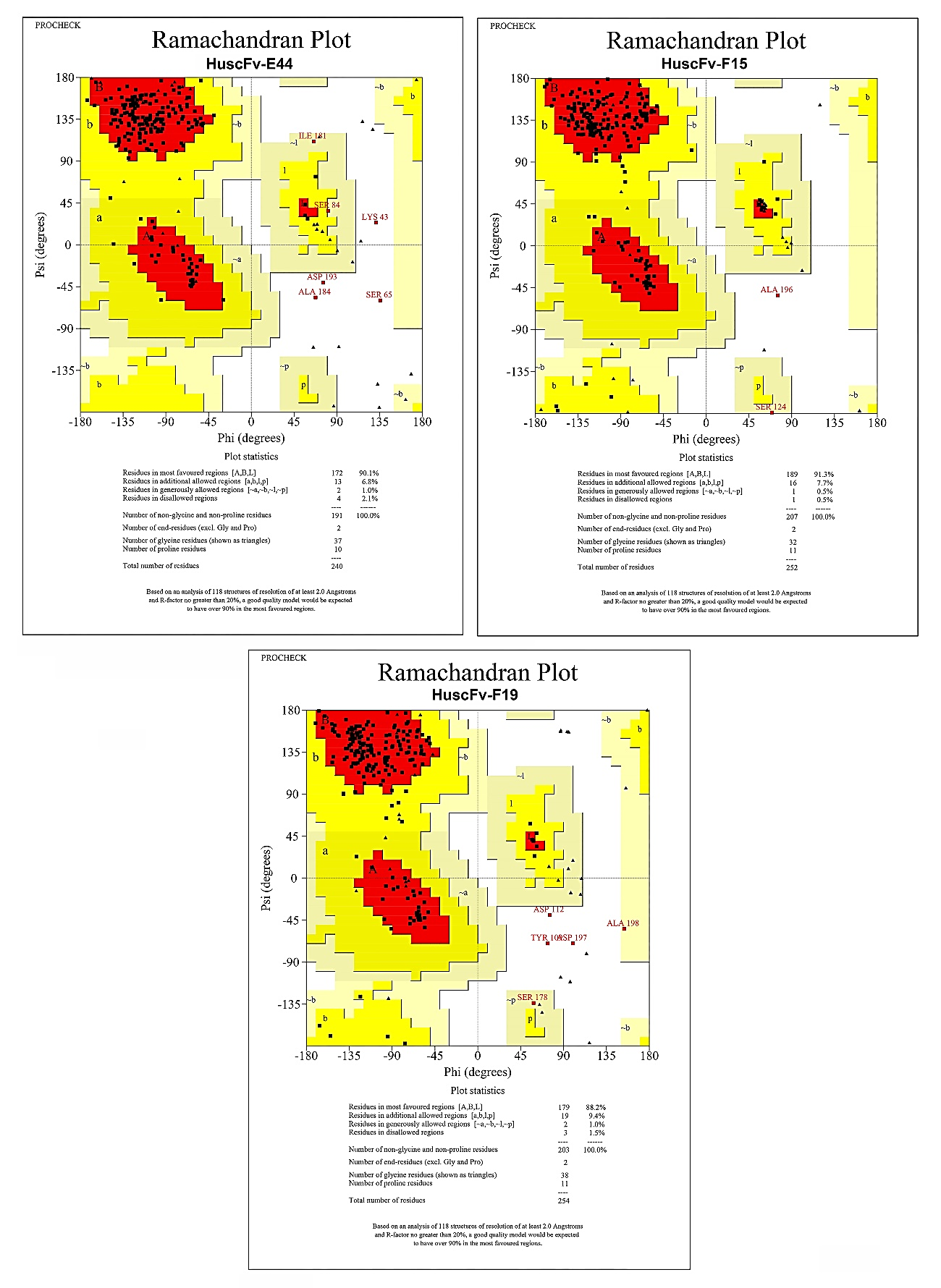
***Supplementary Material***

**SUPPLEMENTARY FIGURE S1 ** Ramachandran plots of the modeled HuscFv-E44, HuscFv-F15 and HuscFv-F19. The most favored regions, additional allowed regions, generously allowed regions and disallowed regions are colored in red, yellow, light yellow and white, respectively.

**
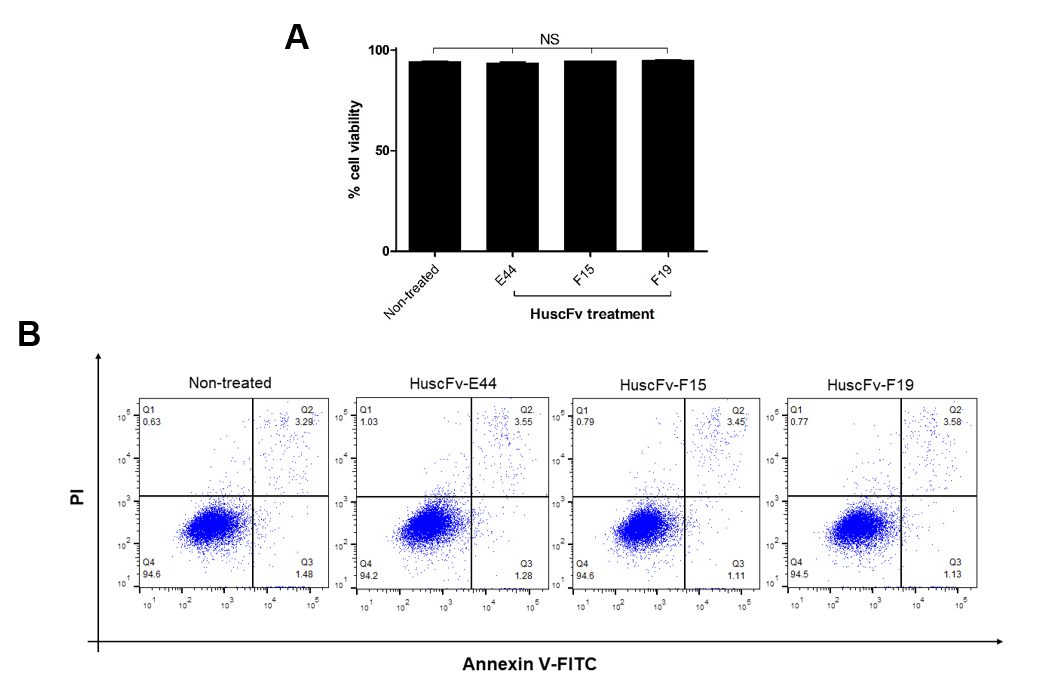
**

**SUPPLEMENTARY FIGURE S2** Biocompatibility of HuscFvs on HeLa cells demonstrated as the percentage of cell viability after treatment with HuscFvs for 24 h. **(A)** The bar graph represents mean ± SD of the percent viability of HeLa cells (n=3). **(B)** Density plots of flow cytometric analysis showing Annexin V/PI stained HuscFv-treated HeLa cells compared with non-treated cells (cells in medium alone) (representative experiment). Percent viable cells (Q4), early apoptotic cells (Q3), late apoptotic cells (Q2) and necrotic cells (Q1).

**
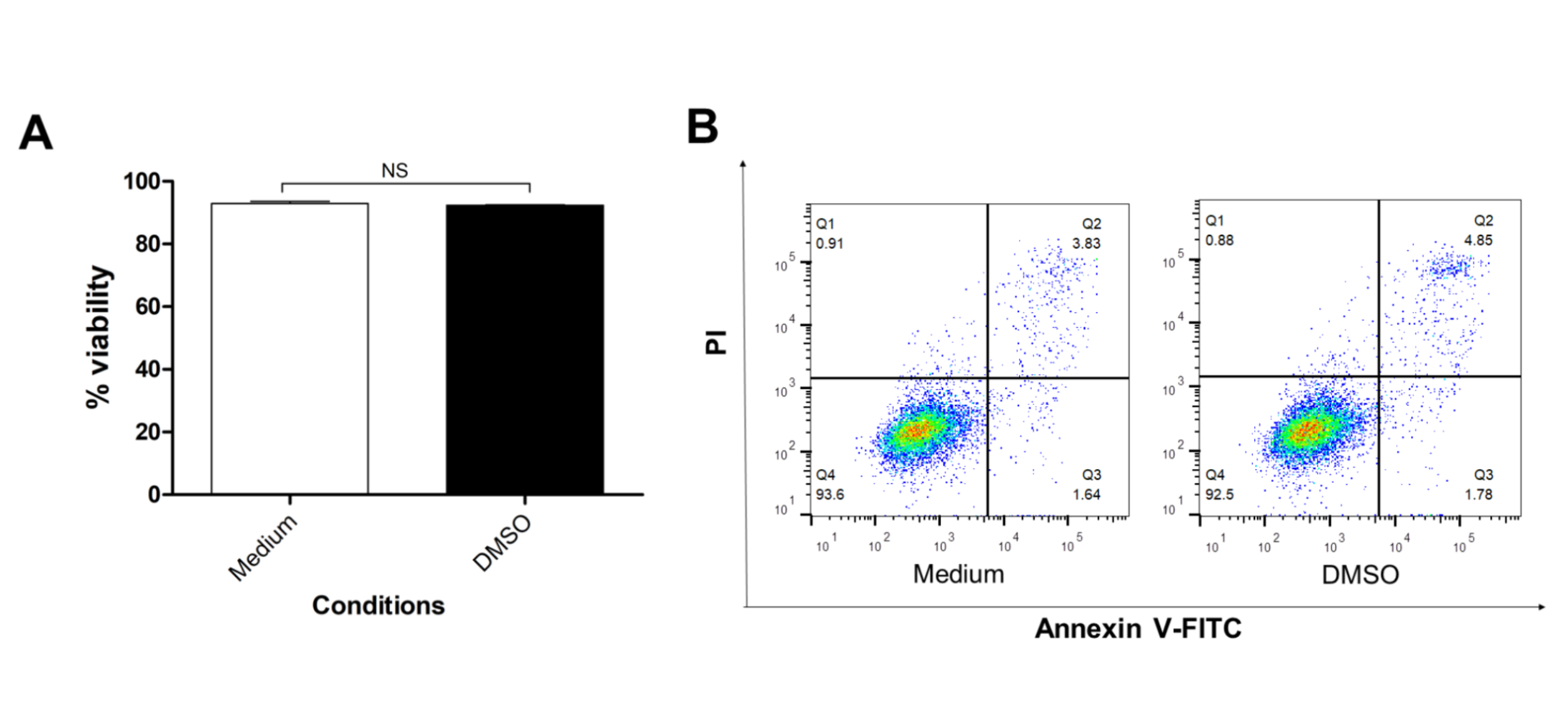
SUPPLEMENTARY FIGURE S3** Percent viability of HeLa cells after exposure to medium containing 0.25% DMSO or medium alone. **(A)** The bar graph represents mean ± SD of the percent HeLa cell viability of both groups. There was no difference (NS) in viability of the cells in medium alone and medium containing the DMSO (*p* > 0.05). **(B)** Representative of density plots of flow cytometric analysis showing Annexin V/PI stained HeLa cells of both groups.

**SUPPLEMENTARY TABLE S1 ** The percentages of overlapping structures between the RS2-1G9 (2NTF) and the HuscFvs-E44, HuscFv-F15, and HuscFv-F19.

| **Proteins** | **Percent overlapped structure** | **RMSD** | **Fragment score** | **Topology score** | **Number of identical residues** |
| --- | --- | --- | --- | --- | --- |
| 2NTF-HuscFv-E44 | 90.83 | 1.53 | 0.96 | 0.52 | 93 |
| 2NTF-HuscFv-F15 | 89.29 | 1.51 | 0.96 | 0.54 | 103 |
| 2NTF-HuscFvF19 | 88.58 | 1.37 | 0.96 | 0.52 | 102 |
